# Ireland DXA-FRAX may differ significantly and substantially to Web-FRAX

**DOI:** 10.1007/s11657-023-01232-y

**Published:** 2023-03-20

**Authors:** Lan Yang, Mary Dempsey, Attracta Brennan, Bryan Whelan, E. Erjiang, Tingyan Wang, Rebecca Egan, Kelly Gorham, Fiona Heaney, Catherine Armstrong, Guadalupe Morote Ibarrola, Amina Gsel, Ming Yu, John J. Carey, the DXA MAP Group

**Affiliations:** 1 Insight SFI Research Centre for Data Analytics, Data Science Institute, University of Galway, IDA Business Park, Lower Dangan, Galway, H91 AEX4 Ireland; 2 School of Engineering, College of Science and Engineering, University of Galway, Galway, Ireland; 3 School of Computer Science, College of Science and Engineering, University of Galway, Galway, Ireland; 4 School of Medicine, College of Medicine, Nursing and Health Sciences, University of Galway, Galway, Ireland; 5 School of Management, Guangxi Minzu University, Nanning, China; 6 Nuffield Department of Medicine, University of Oxford, Oxford, UK; 7 Department of Rheumatology, Galway University Hospitals, Galway, Ireland; 8 Department of Industrial Engineering, Tsinghua University, Beijing, China

**Keywords:** Osteoporosis, Fracture Risk, Bone Mineral Density, FRAX

## Abstract

***Summary*:**

Appropriate use of FRAX reduces the number of people requiring DXA scans, while contemporaneously determining those most at risk. We compared the results of FRAX with and without inclusion of BMD. It suggests clinicians to carefully consider the importance of BMD inclusion in fracture risk estimation or interpretation in individual patients.

**Purpose:**

FRAX is a widely accepted tool to estimate the 10-year risk of hip and major osteoporotic fracture in adults. Prior calibration studies suggest this works similarly with or without the inclusion of bone mineral density (BMD). The purpose of the study is to compare within-subject differences between FRAX estimations derived using DXA and Web software with and without the inclusion of BMD.

**Method:**

A convenience cohort was used for this cross-sectional study, consisting of 1254 men and women aged between 40 and 90 years who had a DXA scan and complete validated data available for analysis. FRAX 10-year estimations for hip and major osteoporotic fracture were calculated using DXA software (DXA-FRAX) and the Web tool (Web-FRAX), with and without BMD. Agreements between estimates within each individual subject were examined using Bland–Altman plots. We performed exploratory analyses of the characteristics of those with very discordant results.

**Results:**

Overall median DXA-FRAX and Web-FRAX 10-year hip and major osteoporotic fracture risk estimations which include BMD are very similar: 2.9% *vs*. 2.8% and 11.0% *vs*. 11% respectively. However, both are significantly lower than those obtained without BMD: 4.9% and 14% respectively, *P* < 0.001. Within-subject differences between hip fracture estimates with and without BMD were < 3% in 57% of cases, between 3 and 6% in 19% of cases, and > 6% in 24% of cases, while for major osteoporotic fractures such differences are < 10% in 82% of cases, between 10 and 20% in 15% of cases, and > 20% in 3% of cases.

**Conclusions:**

Although there is excellent agreement between the Web-FRAX and DXA-FRAX tools when BMD is incorporated, sometimes there are very large differences for individuals between results obtained with and without BMD. Clinicians should carefully consider the importance of BMD inclusion in FRAX estimations when assessing individual patients.

## Background

Osteoporosis and the associated fractures are a major global health burden for patients, their social network, and society [1–3]. Ireland has one of the greatest illness burdens, and the highest projected increases in osteoporotic fractures in Europe over the coming decade [3, 4]. National publications suggest that available data reflect the ‘tip of the iceberg,’ and the financial costs of managing people with these fractures will double during this decade, rising to €2billion by 2030 [5–7]. Recent programs established national standards for the management and audit of hip fracture care among adults aged ≥ 60 years of age and fracture liaison services [8, 9]. These reflect the current state of fragility fracture care, variation in osteoporosis diagnosis, risk assessment and management, some progress, while also highlighting substantial needs including increases in resources, data, policy, priority, and logistics [3, 5–10].

Access to quality risk assessment, diagnosis, and treatment of osteoporosis is heterogeneous and inadequate around the globe [1–3, 11–17]. Many algorithms are available to decide whom to test, how to assess fracture risk, when to intervene, and how to monitor the effects of interventions [1–3, 12, 15, 16, 18–21]. Their performance varies considerably among different populations, with no single method substantially superior to others [18–22]. The Osteoporosis Self-assessment Tool (OST) is one of the simplest algorithms requiring only age and weight to aid in the identification of people likely to have a DXA diagnosis of osteoporosis [18–23]. Appropriate use of the OST index could significantly reduce the number of people requiring a DXA screening test [21–25].

Fracture risk may be estimated using various methods, each with strengths and limitations [2, 18–22]. Substantial efforts over many years supported the development of the FRAX tool such that it has become the dominant fracture risk assessment tool worldwide [26] and is the preferred algorithm of global professional bodies in skeletal health [2, 3, 26–29]. FRAX estimates the 10-year probability of hip fracture (HF) and certain major osteoporotic fractures (MOF) in people aged 40 to 90 years, with country-specific estimate options [26, 29]. Strengths of the FRAX algorithm include the availability of an online calculation option, with or without DXA testing, and the ability to include additional risk factors such as glucocorticoids, secondary causes, and a parental history of hip fracture [26, 29]. This is particularly attractive when access to DXA is limited [14, 20, 26, 29], such as in our center, where reducing unnecessary testing is important [25, 30]. Irish legislation governing the justification of the risks associated with exposure to ionizing radiation prohibits undertaking such exposures if alternative methods are available which can achieve the same objectives [31].

Clinical risk factors included in FRAX are prevalent in Irish adults, including those who are hospitalized and those with and without fractures [17, 32–34]. FRAX probabilities and potential intervention thresholds for Ireland were derived using a limited data set of public hospital admissions, population statistics, and several assumptions, though neither individual-level data nor DXA results were available [4]. Resulting software exists to calculate fracture risk estimates either via the FRAX website (www.sheffield.ac.uk/FRAX/), or on most modern DXA machines. Sometimes we note discordance between FRAX estimates in correspondence we receive and those derived from our DXA machines which include BMD.

The DXA-HIP cohort was established to examine and validate international DXA criteria and osteoporosis diagnostic and prediction algorithms for Irish adults [32]. In order to understand the importance of BMD inclusion when calculating FRAX probabilities for our population, we compare the agreement between Web-based and DXA-based FRAX derivatives for Ireland.

## Methods

Details of the entire DXA-HIP cohort have been described [25, 32]. In brief, a convenience cohort was established for clinical research using DXA data from 3 centers which include 4 GE-Lunar Prodigy DXA machines, using G.E. Encore software version 17. Femoral neck *T*-scores for men and women are generated using NHANES III ISCD-recommended calculations [35]. All scans are performed and reported by staff trained to ISCD standards and recommendations. The staff have regular weekly meetings to discuss discrepancies, complex cases and audit procedures, performance, and reports. The collection and analysis of the data for the DXA-HIP project were approved by our Institutions Ethics Committee and in compliance with G.D.P.R. regulations [25, 32, 36]. In this study, due to the inherent thresholds in the FRAX tool [26], we include Caucasian subjects aged between 40 and 90 years of age with weight less than 125 kg.

Preliminary data for this study were supplied from 1 clinical site between June and December 2021. Data were collected and compiled when auditing our DXA-FRAX estimates at the time of scanning and reporting (G.E. Lunar FRAX estimates for Ireland, version 3.8). Contemporaneously, we derived Web-FRAX estimates for these same men and women from the FRAX website for Ireland (https://www.sheffield.ac.uk/FRAX/, version 4, country code = 48), with and without femoral neck BMD values in g/cm^2^ for GE Lunar. All data were subsequently rechecked on 2 further occasions by 2 of the investigators, and FRAX estimates were recalculated, to ensure the information being used was accurate, consistent, and complete. The data were merged, anonymized, and stored for analysis.

We chose to compare FRAX estimates between DXA-FRAX and Web-FRAX with and without the inclusion of femoral neck BMD for both men and women. In order to highlight the extent and magnitude of the difference between various FRAX estimations, we show the proportion of men and women whose difference between DXA-FRAX with BMD and their corresponding Web-FRAX without BMD HF which were: < 3%, between 3 and 6%, and > 6%, and MOF which were < 10%, between 10 and 20%, and > 20%. We used box and whisker plots and Bland–Altman plots to assess the overall and within-person differences between Web-FRAX with and without BMD and DXA-FRAX and the patterns of bias. We used paired *T*-tests, Chi-squared tests, Fisher’s exact tests, and Wilcoxon Rank Sum tests to compare means and medians as appropriate. All analyses were planned ad hoc. All analyses were performed on Python 3.6. We performed sensitivity analyses by excluding those whose prior fracture site was unknown, and for those with multiple prior fragility fractures.

## Results

A total of 2090 records were collected during an audit of vertebral fracture assessment (VFA) scans between 2019 and 2021 including patients’ demographic information such as age, gender, weight, height, and BMI; risk factors such as previous fracture and femoral neck BMD; and results of DXA-FRAX estimations. Subjects with missing or incomplete information were excluded. Complete data on 1254 adults aged between 40 and 90 years were available for this study, including 290 (23.1%) men and 964 (76.9%) women. A summary of patient details including the variables used in FRAX calculations is shown in Table 1, broken down by gender. Women are significantly lighter and shorter than men, had lower BMD, and were less likely to take corticosteroids or drink excessively, but more likely to have a parent who had a hip fracture. Almost half of the men and women had a previous MOF, while almost 36% have another disorder strongly associated with osteoporosis such as early menopause, diabetes mellitus, or coeliac disease.

**Table 1:** Characteristics of study subjects

	Female	Male	*P* value
Number of subjects	964	290	
Mean age in years (SD)	68.5 (9.7)	69.2 (11.4)	0.38
Mean weight in kg (SD)	69.1 (14.4)	83.3 (15.4)	< 0.001
Mean height in m (SD)	1.6 (0.1)	1.7 (0.1)	< 0.001
Mean body mass index (kg/m^2^) (SD)	27.1 (5.4)	28.0 (4.8)	< 0.001
Prevalent fracture, *N* (%)	458 (47.5)	141 (48.6)	0.79
Parent fractured hip, *N* (%)	71 (7.4)	6 (2.1)	< 0.001
Current smokers, *N* (%)	91 (9.4)	33 (11.4)	0.39
Current glucocorticoid use, *N* (%)	132 (13.7)	116 (40.0)	< 0.001
Rheumatoid arthritis, *N* (%)	106 (11.0)	32 (11.0)	1.00
Secondary osteoporosis, *N* (%)	344 (35.7)	103 (35.5)	1.00
Alcohol ≥ 3 units/day, *N* (%)	5 (0.5)	10 (3.4)	< 0.001
Mean femoral neck *T*-score (SD)	− 1.6 (0.8)	− 1.0 (1.1)	< 0.001

**Fig. 1:**
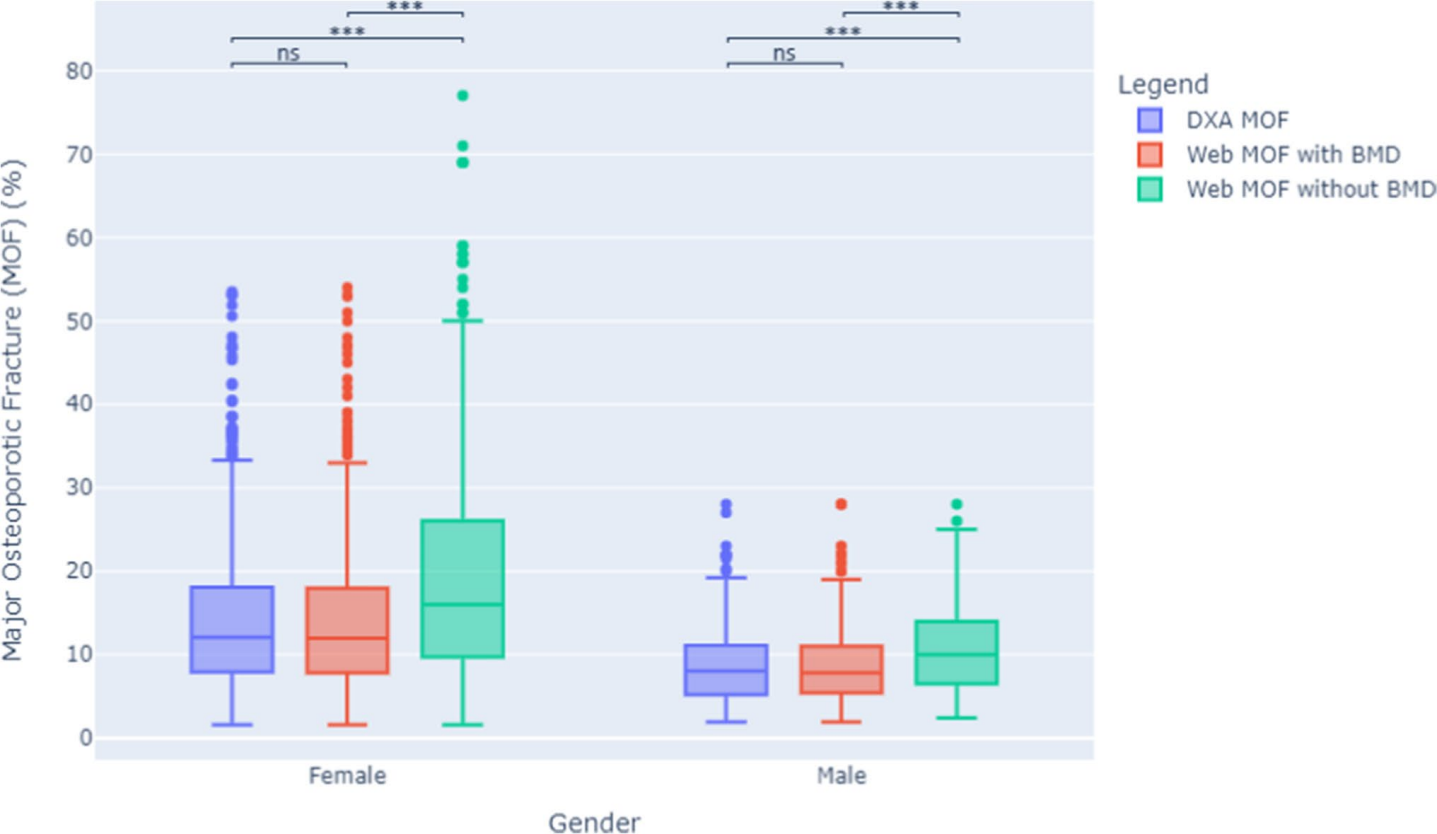
Box and whisker plots comparing DXA-FRAX, Web-FRAX with BMD, and Web-FRAX without BMD for MOF by gender. Note: the significance is reported for the following levels: ns: not significant; ^*^P < 0.05, ^**^P < 0.01, and ^***^P < 0.001

**Fig. 2:**
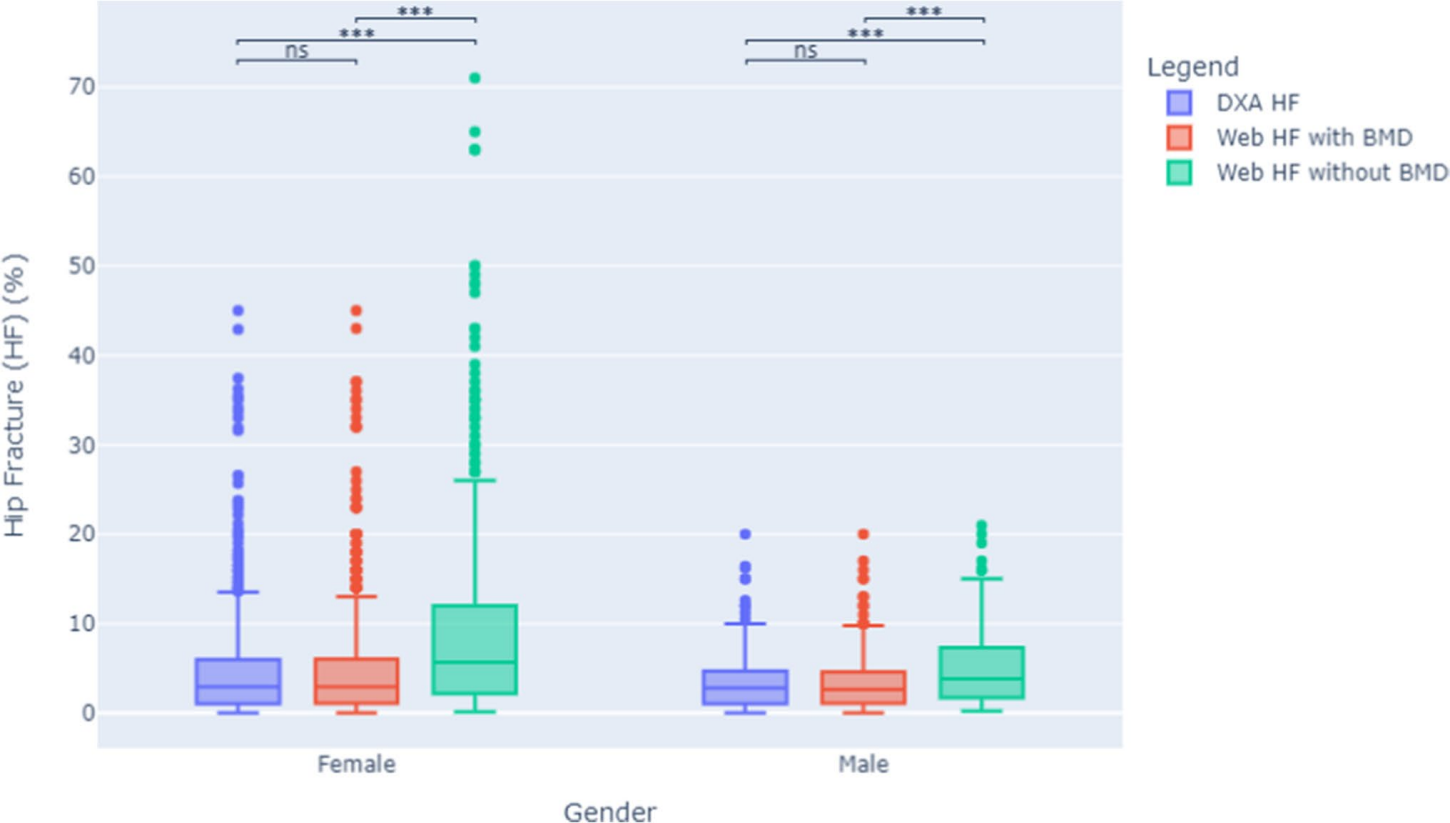
Box and whisker plots comparing DXA-FRAX, Web-FRAX with BMD, and Web-FRAX without BMD for HF by gender. Note: the significance is reported for the following levels: ns: not significant; ^*^P < 0.05, ^**^P < 0.01, and ^***^P < 0.001

**Fig. 3:**
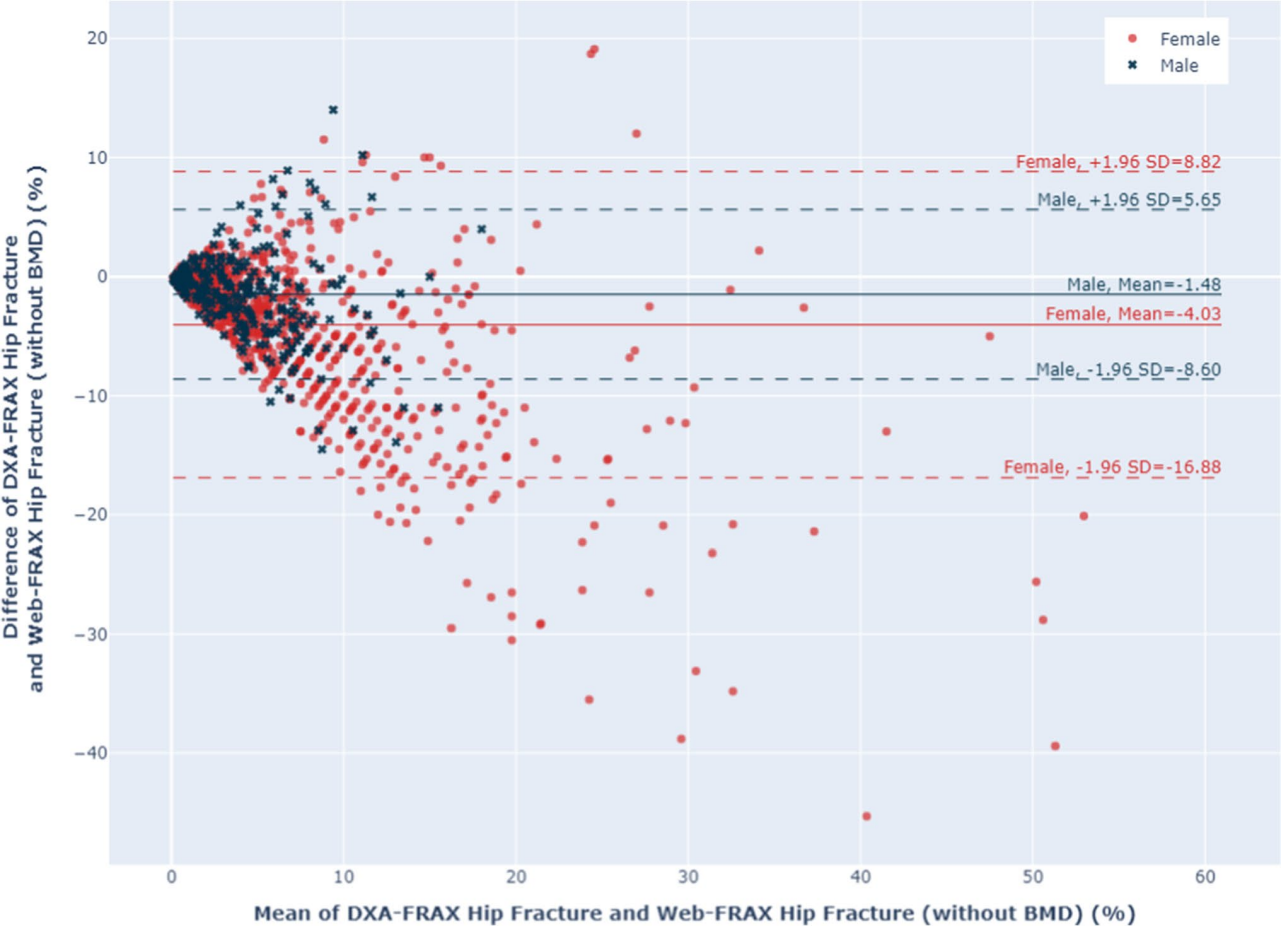
Bland–Altman plots comparing DXA-FRAX to Web-FRAX without BMD for HF for by gender

**Fig. 4:**
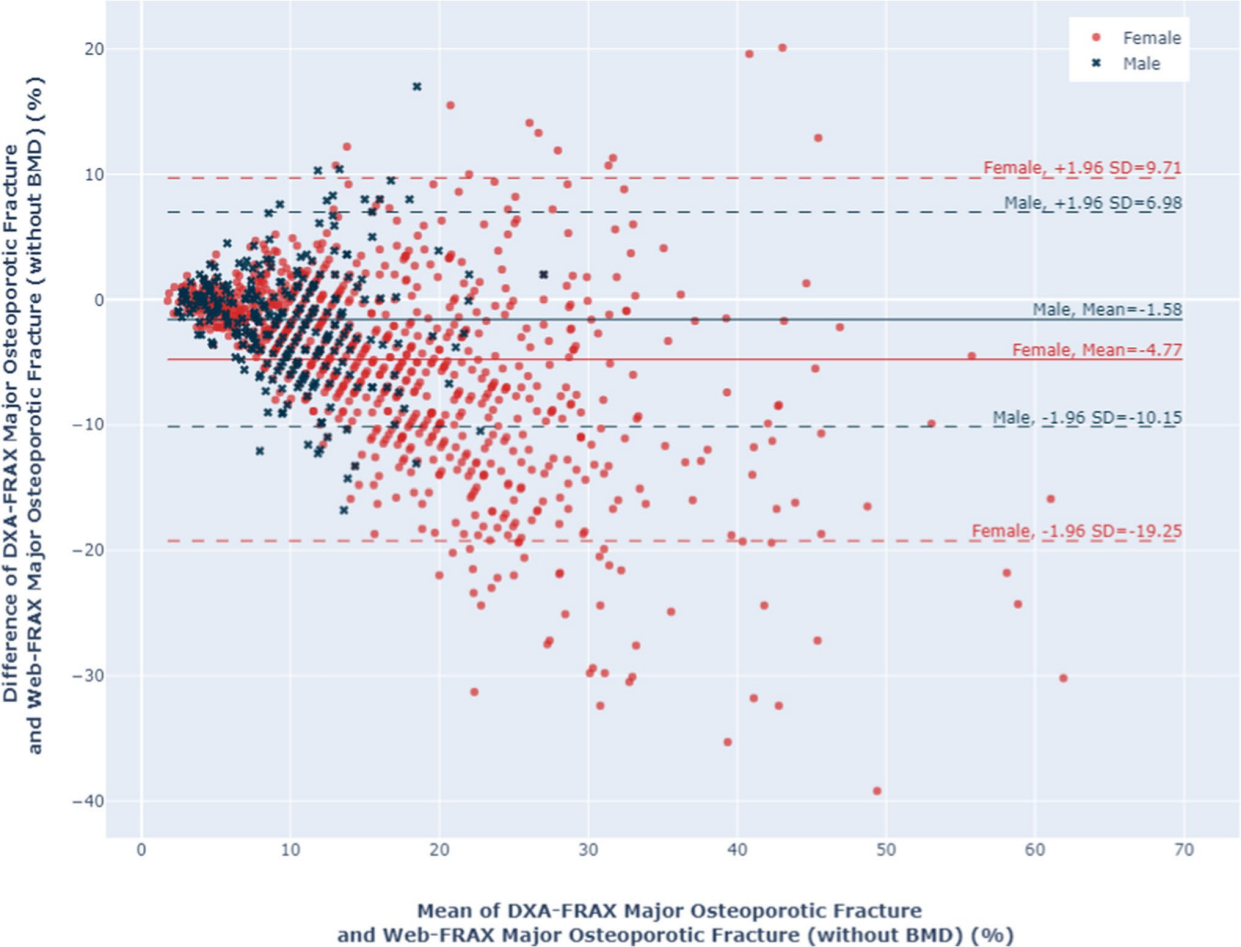
Bland–Altman plots comparing DXA-FRAX to Web-FRAX without BMD for MOF by gender

Women had higher FRAX scores than men using all 3 calculation methods, for both HF and MOF, shown in Figs. 1 and 2. The majority of men and women had DXA-FRAX HF scores below 5% and MOF less than 20%. A small number of female patients have very high scores (> 50%) for both HF and MOF. Overall, Web-FRAX scores without BMD were significantly higher, *P* < 0.001, than Web-FRAX scores with BMD or DXA-FRAX scores for both men and women, and both HF and MOF, shown in Table 2, though for individuals they were sometimes lower, Figs. 3 and 4. Differences between DXA-FRAX and Web-FRAX with BMD were very small and not statistically significant (*P* values HF: 0.914, MOF: 0.967) shown in Appendix Figs. 5 and 6, whereas the differences between DXA-FRAX and Web-FRAX without BMD as well as the differences between Web-FRAX with BMD and Web-FRAX without BMD are sometimes large and were statistically significant, ^***^P < 0.001 (Figs. 3 and 4). The prevalence of hip fracture and major osteoporotic fracture for women is 3.4% for HF and 47.5% for MOF, while for men is 6.6% for HF and 48.6% for MOF.

**Table 2:** Comparison of DXA-FRAX and Web-FRAX scores with and without BMD for MOF and HF

Gender	Fracture risk	DXA-FRAX	Web-FRAX with BMD	Web-FRAX without BMD
Female	Median MOF (IQR)	12.1 (7.9–18.1)	12.0 (7.8–18.0)	16.0 (9.7–26.0)
	Median HF (IQR)	2.9 (1.0–6.0)	2.9 (1.1–6.0)	5.7 (2.2–12.0)
Male	Median MOF (IQR)	8.0 (5.3–11.1)	7.8 (5.4–11.0)	10.0 (6.5–14.0)
	Median HF (IQR)	2.8 (1.0–4.7)	2.6 (1.1–4.6)	3.8 (1.7–7.3)
All	Median MOF (IQR)	11.0 (7.0–16.2)	11.0 (7.0–16.0)	14.0 (8.5–23.0)
	Median HF (IQR)	2.9 (1.0–5.5)	2.8 (1.1– 5.5)	4.9 (2.0–10.0)

In contrast, we found substantial differences within individuals when we compared the absolute difference in HF and MOF between their DXA-FRAX and their Web-FRAX scores without BMD, particularly among those with higher scores, as shown in the Bland–Altman plots in Figs. 3 and 4. However, for those with low scores: HF < 5% and MOF < 10%, the absolute differences were generally small. A similar pattern was noted when we compared Web-FRAX scores with BMD to Web-FRAX scores without BMD, data not shown. The differences observed between DXA-FRAX and the corresponding Web-FRAX with BMD estimates for HF and MOF were generally very small, with limits of agreement of < 1% and maximum differences of < 2.4%, shown in Appendix Figs. 5 and 6. Table 3 presents the breakdown of the proportion of individuals with various differences between DXA-FRAX and Web-FRAX scores without BMD, HF: < 3%, 3 to 6%, and > 6%, and MOF: < 10%, 10–20%, and > 20% difference. These show a greater proportion of women have larger absolute differences > 10% than men for MOF. However, 43% of the patients have an absolute difference in HF of > 3%. Moreover, 28% of females and 14% of males have an absolute difference in HF estimates of > 6%. The range of differences for women is 0–39.2% for MOF and 0–45.3% for HF while for men is 0–17.0% for MOF and 0–14.5% for HF.

**Table 3:** Proportion of individuals with various differences in HF and MOF between DXA-FRAX and Web-FRAX without BMD scores

Gender	Hip fracture			Major osteoporotic fracture		
	|*x*|< 3%	3 ≤|*x*|< 6%	|*x*|≥ 6%	|*x*|< 10%	10% ≤|*x*|< 20%	|*x*|> 20%
Female	524 (54%)	174 (18%)	266 (28%)	757 (78%)	170 (18%)	37 (4%)
Male	190 (65%)	60 (21%)	40 (14%)	275 (95%)	15 (5%)	0 (0%)
All	714 (57%)	234 (19%)	306 (24%)	1032 (82%)	185 (15%)	37 (3%)

*x* indicates DXA-FRAX score minus Web-FRAX without BMD score

Table 4 summarizes the characteristics of people whose DXA-FRAX and corresponding Web-FRAX without BMD scores differ by a small, moderate, or large amount. Fractures, secondary osteoporosis, and rheumatoid arthritis were more prevalent among those with larger differences, who are also older, lighter, and have lower BMI. In Tables 5 and 6, we summarize the characteristics of those women and men, respectively, whose differences between their DXA-FRAX and Web-FRAX scores were greater than or less than the limits of agreement derived from our Bland–Altman results (Figs. 3 and 4). Women with more extreme differences were older and lighter and had lower BMI and BMD; a greater prevalence of fractures, rheumatoid arthritis, and glucocorticoid use; and a much higher or lower prevalence of secondary osteoporosis, tobacco use, or a parent with a previous hip fracture. Men with more extreme differences were similarly lighter and had a lower BMI, a greater prevalence of fractures and excessive alcohol use, a lower prevalence of parents with a hip fracture, a higher or lower age, BMD, and prevalence of smoking, glucocorticoid use, rheumatoid arthritis, and secondary osteoporosis.

**Table 4:** Characteristics of individuals with various differences in HF and MOF between DXA-FRAX and Web-FRAX without BMD scores

	Hip fracture			Major osteoporotic fracture		
	|*x*|< 3%	3 ≤|*x*|< 6%	|*x*|≥ 6%	|*x*|< 10%	10% ≤|*x*|< 20%	|*x*|> 20%
Number of subjects	713	233	308	1031	186	37
Mean age in years (SD)	64.0 (9.0)	72.3 (7.4)	76.7 (7.9)	66.8 (9.7)	76.7 (6.9)	80.5 (4.9)
Mean weight in kg (SD)	76.4 (16.2)	71.0 (13.2)	64.2 (13.1)	74.4 (15.8)	63.9 (12.7)	60.0 (10.0)
Mean height in m (SD)	1.6 (0.1)	1.6 (0.1)	1.6 (0.1)	1.6 (0.1)	1.6 (0.1)	1.6 (0.1)
Mean BMI in kg/m^2^ (SD)	28.4 (5.3)	26.9 (4.5)	25.0 (5.0)	27.8 (5.2)	25.3 (4.9)	23.9 (3.9)
Prevalent fracture, *N* (%)	259 (36.3)	121 (51.9)	219 (71.1)	439 (42.6)	124 (66.7)	36 (97.3)
Parent fractured hip, *N* (%)	44 (6.2)	14 (6.0)	19 (6.2)	60 (5.8)	11 (5.9)	6 (16.2)
Current smokers, *N* (%)	67 (9.4)	24 (10.3)	33 (10.7)	102 (9.9)	15 (8.1)	7 (18.9)
Current glucocorticoid use, *N* (%)	141 (19.8)	45 (19.3)	62 (20.1)	204 (19.8)	39 (21.0)	5 (13.5)
Rheumatoid arthritis, *N* (%)	63 (8.8)	30 (12.9)	45 (14.6)	105 (10.2)	24 (12.9)	9 (24.3)
Secondary osteoporosis, *N* (%)	201 (28.2)	98 (42.1)	148 (48.1)	331 (32.1)	93 (50.0)	23 (62.2)
Alcohol ≥ 3 units/day, *N* (%)	3 (0.4)	7 (3.0)	5 (1.6)	12 (1.2)	2 (1.1)	1 (2.7)
Mean femoral neck *T*-score (SD)	− 1.3 (0.9)	− 1.5 (0.9)	− 1.6 (1.0)	− 1.4 (0.9)	− 1.6 (1.0)	− 1.2 (0.8)

*x* indicates DXA-FRAX score minus Web-FRAX without BMD score

**Table 5:** Comparison of extreme differences between DXA-FRAX and Web-FRAX for women

	Hip fracture		Major osteoporotic f09racture	
	*x* > 8.82%	*x* < − 16.88%	*x* > 9.71%	*x* < − 9.25%
Number of subjects	9	44	12	42
Mean age in years (SD)	70.3 (9.1)	79.8 (5.3)	69.4 (7.9)	80.2 (5.1)
Mean weight in kg (SD)	60.5 (10.3)	55.4 (9.3)	70.5 (22.8)	59.8 (10.5)
Mean height in m (SD)	1.6 (0.1)	1.6 (0.1)	1.6 (0.1)	1.6 (0.1)
Mean BMI in kg/m^2^ (SD)	24.8 (4.7)	22.1 (3.1)	29.0 (9.8)	23.7 (4.0)
Prevalent fracture, *N* (%)	8 (88.9)	36 (81.8)	10 (83.3)	39 (92.9)
Parent fractured hip, *N* (%)	0 (0)	11 (25.0)	1 (8.3)	7 (16.7)
Current smokers, *N* (%)	0 (0)	8 (18.2)	1 (8.3)	8 (19.0)
Current glucocorticoid use, *N* (%)	2 (22.2)	14 (31.8)	3 (25.0)	8 (19.0)
Rheumatoid arthritis, *N* (%)	2 (22.2)	11 (25.0)	3 (25.0)	8 (19.0)
Secondary osteoporosis, *N* (%)	2 (22.2)	22 (50.0)	1 (8.3)	27 (64.3)
Alcohol ≥ 3 units/day, *N* (%)	0 (0)	1 (2.3)	0 (0)	1 (2.4)
Mean femoral neck t-score (SD)	-3.5 (0.3)	-1.6 (0.8)	-3.3 (0.4)	-1.3 (0.7)

(1) extreme differences: indicates subjects whose FRAX estimates differ by values which exceed (above or below) the limits of agreement line on the Bland–Altman plots. (2) *x* indicates DXA-FRAX score minus Web-FRAX without BMD score

**Table 6:** Comparison of extreme differences between DXA-FRAX and Web-FRAX for men

	Hip fracture		Major osteoporotic fracture	
	*x* > 5.65%	*x* < − 8.60%	*x* > 6.98%	*x* < − 10.15%
Number of subjects	11	10	11	11
Mean age in years (SD)	59.5 (8.8)	82.2 (3.2)	61.9 (7.4)	80.0 (4.5)
Mean weight in kg (SD)	70.0 (15.6)	67.5 (12.8)	73.8 (15.4)	69.9 (12.2)
Mean height in m (SD)	1.8 (0.1)	1.7 (0.1)	1.8 (0.1)	1.7 (0.1)
Mean BMI in kg/m^2^ (SD)	22.6 (4.4)	23.2 (3.1)	24.0 (4.9)	23.9 (3.3)
Prevalent fracture, *N* (%)	10 (90.9)	5 (50.0)	10 (90.9)	7 (63.6)
Parent fractured hip, *N* (%)	0 (0)	0 (0)	0 (0)	0 (0)
Current smokers, *N* (%)	3 (27.3)	0 (0)	3 (27.3)	0 (0)
Current glucocorticoid use, *N* (%)	3 (27.3)	7 (70.0)	2 (18.2)	6 (54.5)
Rheumatoid arthritis, *N* (%)	0 (0)	3 (30.0)	0 (0)	2 (18.2)
Secondary osteoporosis, *N* (%)	3 (27.3)	7 (70.0)	2 (18.2)	8 (72.7)
Alcohol ≥ 3 units/day, *N* (%)	3 (27.3)	1 (10.0)	3 (27.3)	1 (9.1)
Mean femoral neck *T*-score (SD)	− 2.8 (0.4)	− 0.4 (1.7)	− 2.8 (0.4)	− 0.0 (1.3)

(1) extreme differences: indicates subjects whose FRAX estimates differ by values which exceed (above or below) the limits of agreement line on the Bland–Altman plots. (2) *x* indicates DXA-FRAX score minus Web-FRAX without BMD score

## Discussion

In this paper comparing different FRAX calculations in older Irish men and women, we found excellent agreement between the Web version and the DXA version when femoral neck BMD was included. However, when we compared estimations without BMD to estimations that included BMD, there were notable differences for some individuals or extreme cases which at times were quite large, up to 40% absolute difference for major osteoporotic fracture and 46% absolute difference for hip fracture, shown in Figs. 3 and 4. Such differences are more likely to be observed at extremes of weight, BMI, BMD, or prevalence of rheumatoid arthritis or secondary causes of osteoporosis, as well as where fractures or glucocorticoid use is present.

FRAX is a clinical tool designed to improve the estimation of fracture risk by combining some of the most important determinants in a multivariate algorithm, which should be more robust than using any single factor [29, 37–40]. Additionally, the importance of using absolute risk rather than relative risk or a single BMD threshold is an important advance [26, 29]. The original algorithm was derived from 9 cohorts including 46,340 men (32%) and women (68%) and validated across 11 cohorts including 230,486 men (< 1%) and women (> 99%) from 23 countries across the globe [37]. BMD was available for 37,305 (80.5%) of the development group, but only 28,660 (12.4%) of the validation group [37]. This algorithm is now available in a number of formats, including a web-based calculator and a DXA-based calculator [39, 41]. The current web-based tool (31^st^ October 2022) includes 87 populations: 18 Asian populations, 36 European populations, 19 Middle East and African Populations, 5 North American populations, 7 Latin American populations, and 2 Oceania populations, while the DXA-based tool has 58 available populations to select from. Previous attempts to calibrate FRAX for Ireland used national hip fracture estimates but no patient-level data, and the authors note the inclusion of BMD could be problematic [4]. Today, FRAX is widely used in the assessment of individuals, despite the lack of validation within a large representative population [3]. Since the FRAX tool has been incorporated in over 80 guidelines worldwide [39, 42], there is a need for assurances of accuracy and consistency in outputs.

Several authors compare the performance of the tool using different calculation methods, with and without BMD, and to other risk algorithms, showing variability within and between populations [33, 37, 41, 43–52]. Some suggest FRAX performs similarly with and without BMD [43, 46, 49, 50], and using different calculation methods [41], while others suggest FRAX without BMD is not sensitive enough to identify those in need of treatment [33, 46–48, 51, 52]. A Japanese study comparing 4 different FRAX calculation methods for several thousand men and women show they provide similar estimates [41], while a group of Danish authors suggests the addition of BMD may be of limited benefit [43]. In our study, the inclusion of BMD reduces the mean FRAX Ireland estimates for both 10-year risk of HF and MOF, for both men and women. More importantly, for some individuals, there were very large differences when BMD was included in their calculation. Such differences could have a significant influence on patient and clinician decisions on whether, and how, to intervene or not, and the downstream clinical consequences for the patient in terms of benefit, risk, and cost.

Prior studies compare FRAX estimates with and without BMD using ROC (receiver operatic characteristic) curves and AUC (area under the curve) analyses, or a comparison of means [37, 44–46, 49–51]. ROC analysis is commonly used to assess the accuracy of diagnostic testing, but has important limitations, particularly when examining risk [53, 54]. Common errors in the medical literature include interpreting comparisons between two effects without directly comparing them, and over-interpreting non-significant results [55]. Inevitably, there will be differences between measures when different methods are applied; hence, the key issue is really the quantity of these differences [56]. In our study, the AUC values obtained with and without BMD are similar in pattern to prior publications whereby the inclusion of BMD improved the value. Unlike other studies, the AUC for MOF was greater than the AUC for hip fractures, likely due to the overfitting of the model with a very high fracture prevalence, particularly non-hip MOF in our sample. In a sensitivity analysis where we excluded those with multiple fractures or missing fracture sites, this provided a marginal improvement. However, the key aim of the study was to examine the within-person difference in FRAX estimates for different calculation methods. A more formal analysis of the differences between estimations within individuals displays a far more accurate picture of the size of the problem, and where those problems tend to arise. We also show when such differences are more likely to be seen. It would appear from our data that use of the FRAX tool without BMD should be interpreted cautiously for individual patients, especially older patients or those deemed higher risk.

Our study has important limitations. Firstly, these data represent a small sample of a larger dataset, but this analysis is an important first step in a multi-step process to examine and understand the validity of FRAX and other tools for our population with and without BMD. Secondly, the data are cross-sectional in nature, so while we can use the tool to estimate risk, and discriminate between those with and without prevalent fracture, we cannot calibrate the results. These results are important however as such assessments with and without BMD are in widespread use in clinical practice today in Ireland. Thirdly, all subjects were referred for a DXA scan for a reason and almost 50% have a prevalent fracture, so these results may not apply to a more general population, or those without prior fractures. Current studies are assessing the performance among those with and without risk factors, and with and without prior fractures in the larger dataset, and longitudinal analyses to calibrate this and other risk algorithms in a larger cohort. Our larger dataset is incomplete and has some missing data, but this small subset represents a sample that has been triple-checked for the accuracy and completeness of the data for all study subjects enabling a more robust comparison. Finally, there are many different versions of the FRAX tool in use today, and our results may not apply to other populations where the importance of BMD has been clarified or remains unknown.

## Conclusions

Significant differences exist in the results of DXA-FRAX and Web-FRAX for Ireland, particularly for men and those with higher risk estimates so these results should be interpreted cautiously. Reassuringly, results were similar for those deemed at lower risk and for women. These results support the need for a more formal longitudinal analysis to calibrate FRAX and other risk tools for our population, with and without BMD.

## Figures and Tables

**Fig. 5:**
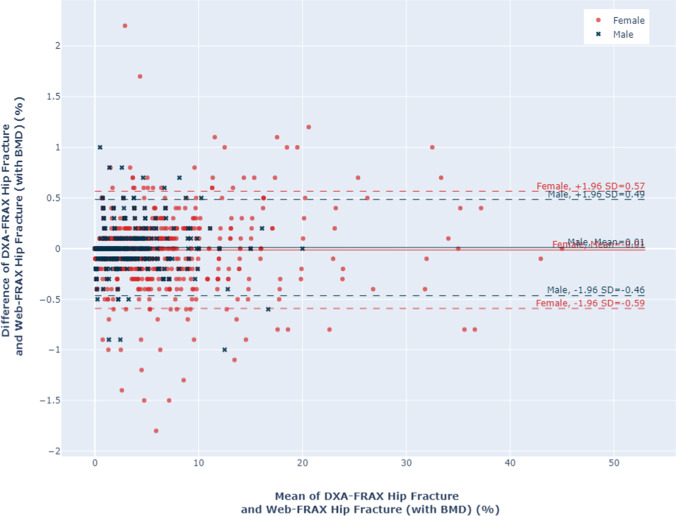
Bland–Altman plots comparing DXA-FRAX to Web-FRAX with BMD for HF by gender

**Fig. 6:**
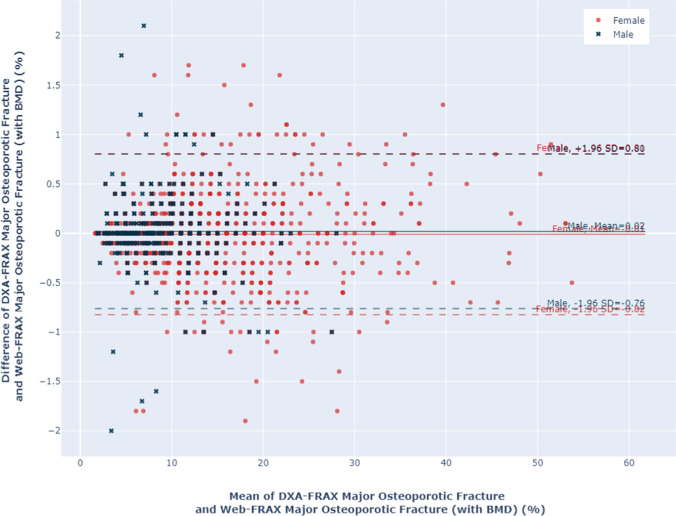
Bland–Altman plots comparing DXA-FRAX to Web-FRAX with BMD for MOF by gender
